# Sulforaphene Ameliorates Neuroinflammation and Hyperphosphorylated Tau Protein via Regulating the PI3K/Akt/GSK-3*β* Pathway in Experimental Models of Alzheimer's Disease

**DOI:** 10.1155/2020/4754195

**Published:** 2020-09-10

**Authors:** Wen Yang, Yue Liu, Qing-Qing Xu, Yan-Fang Xian, Zhi-Xiu Lin

**Affiliations:** ^1^School of Chinese Medicine, Faculty of Medicine, The Chinese University of Hong Kong, Shatin, N.T., Hong Kong SAR, China; ^2^Cardiovascular Disease Centre, Xiyuan Hospital of China Academy of Chinese Medical Sciences, Beijing, China; ^3^Brain Research Centre, School of Chinese Medicine, Faculty of Medicine, The Chinese University of Hong Kong, Hong Kong SAR, China; ^4^Hong Kong Institute of Integrative Medicine, The Chinese University of Hong Kong, Hong Kong SAR, China

## Abstract

Alzheimer's disease (AD) is the most common form of dementia characterized by progressive loss of cognitive functions due to neuronal death mainly in the hippocampal and cortical brain. Sulforaphene (SF) is one of the main isothiocyanates isolated from a Chinese herb Raphani Semen. In this study, we aimed to investigate the neuroprotective effects of SF using *in vitro* and *in vivo* models of AD. Streptozotocin (STZ) was intracranially injected into the rats; then, SF (25 and 50 mg/kg) was given orally once a day for 6 consecutive weeks. After drug treatment, the cognitive functions were assessed using the Morris Water Maze Test (MWMT). After the MWMT, the rats were euthanized and brain tissues were collected. In the *in vitro* test, BV-2 microglia were pretreated with SF (1 and 2 *μ*M) for 1 h and then stimulated with lipopolysaccharide (LPS) for another 23 h. Both molecular and histological methods were used to unravel the action mechanisms and elucidate the signaling pathway. The MWMT results showed that SF treatment significantly improved the STZ-induced cognitive deficits in rats. SF treatment markedly suppressed the production of tumor necrosis factor-*α* (TNF-*α*) and interleukin-6 (IL-6) but increased the release of IL-10 in the STZ-treated rats. In addition, SF significantly inhibited the phosphorylation of tau protein at Thr205, Ser396, and Ser404 sites, while enhancing the ratios of p-Akt (Ser473)/Akt and p-GSK-3*β* (Ser9)/GSK-3*β* in the hippocampus of the STZ-treated rats. On the other hand, SF (1 and 2 *μ*M) treatment also markedly attenuated the cytotoxicity induced by LPS in BV-2 cells. In addition, SF treatment obviously suppressed the releases of nitric oxide (NO), TNF-*α*, and IL-6 in the LPS-stimulated BV-2 cells. Moreover, SF treatment significantly mitigated the nuclear translocation of p-NF-*κ*B p65 and the ratio of p-GSK-3*β* (Ser9)/GSK-3*β* in LPS-stimulated BV-2 cells. Taken together, SF possessed neuroprotective effects against the STZ-induced cognitive deficits in rats and LPS-induced neuroinflammation in BV-2 cells via modulation of the PI3K/Akt/GSK-3*β* pathway and inhibition of the NF-*κ*B activation, suggesting that SF is a promising neuroprotective agent worthy of further development into AD treatment.

## 1. Introduction

Alzheimer's disease (AD) is a progressive neurodegenerative disorder characterized by dysfunction of synapses and loss of neurons in the cerebral cortex and hippocampus of the brain. Clinically, AD usually causes cognitive deficits and memory loss. Despite the complex pathogenesis of AD, the two major neuropathological hallmarks of AD brain are the formation of extracellular senile plaques from beta amyloids (A*β*) and intracellular neurofibrillary tangles (NFTs) composed of hyperphosphorylated tau [1]. Increasing evidence indicates that neuroinflammation is intimately involved in the AD pathogenesis such as amyloid deposition, neuronal damage, tangle formation, and neuronal death. Neuroinflammation occurs at a very early stage of AD, even before the formation of A*β* plaques and tangles. Under inflammatory condition, microglial cells are activated, and the production of proinflammatory cytokines such as interleukin-6 (IL-6), tumor necrosis factor-*α* (TNF-*α*), IL-1, and interferon-*γ* (IFN-*γ*) is enhanced. Activated microglia and neuroinflammation could cause oxidative stress and neuronal damage, leading to amyloid plaque and tangle formation in the brain of AD patients [2]. Thus, the identification of therapeutic agents that can regulate neuroinflammation would be a plausible way in AD prevention and treatment.

A growing body of evidence suggests that intracerebroventricular (*i.c.v.*) injections of streptozotocin (STZ) in a rat are a feasible and workable model that can break down the insulin pathway and cause chronic inflammation and lead to the progressive deficits in learning, memory, and other cognitive behaviors [3, 4]. This animal model also features neuroinflammation, the accumulation of tangles and amyloid plaques, and oxidative stress [5–7]. Therefore, *i.c.v.* STZ administration has been extensively used as a rodent experimental model for studies of sporadic AD [8].

**Figure 1:**
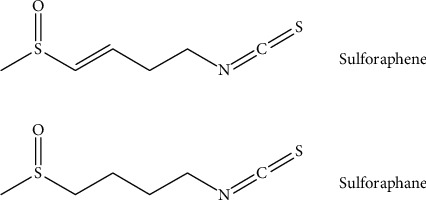
The chemical structures of sulforaphane (SFN) and sulforaphene (SF) (explains briefly the chemical class of these compounds).

Isothiocyanates (ITCs) are phytochemicals derived from cruciferous (*Brassicaceae*) plants characterized by the presence of an isothiocyanate (-N=C=S) moiety in the chemical structure [9]. ITCs have been reported to possess a variety of pharmacological activities such as antioxidant, anti-inflammatory, anticarcinogenic actions, and neuroprotective effects. Sulforaphane (SFN), an ITC enriched in broccoli, has been reported to exert anti-AD effects in several animal models of AD [10–14]. SFN has been considered to be a promising anti-AD agent. Sulforaphene (SF) (chemical structures of SF and SFN are shown in Figure 1) is one of the main isothiocyanates isolated from Raphani Semen (Lai-Fu-Zi in Chinese) [15]. The chemical structures of SF and SFN are very similar. SF is distinguished from SFN by having a double bond between the third and fourth carbon. Studies have reported that the content of SF in Raphani Semen is 7 mg/g which is much higher than SFN (77.19-89.19 *μ*g/g) [16]. Moreover, the extraction process of SF from Raphani Semen is much easier than that of SFN [17]. Therefore, we hypothesized that SF may have potential anti-AD effects in experimental models of AD. In this study, *i.c.v.* injection of STZ into the rats and the lipopolysaccharide- (LPS-) stimulated BV-2 microglial cells were used as *in vivo* and *in vitro* models of AD to investigate the anti-AD effects of SF. The Morris Water Maze Test (MWMT) was employed to assess the functions of spatial learning and memory. Moreover, the effects of SF on neuroinflammation, hyperphosphorylation of tau protein, and the PI3K/Akt/GSK-3*β* pathway were determined to illustrate the neuroprotective action mechanism of SF.

## 2. Materials and Methods

### 2.1. Chemicals and Reagents

Sulforaphene (SF, purity ≥ 95%) was obtained from Chengdu Biopurify Pharmaceutical Co. Ltd. (Chengdu, Sichuan, China), and its identity was confirmed by comparing its ^1^H NMR and ^13^C NMR spectra with those published in the literature [17]. Donepezil hydrochloride, lipopolysaccharide, LY294002, and 3-(4,5-dimethyl-2-thiazolyl)-2,5-diphenyl-2-H-tetrazolium bromide (MTT) were purchased from Sigma-Aldrich (St. Louis, MO, USA). STZ was purchased from Santa Cruz Biotechnology (Dallas, USA). All other reagents and chemicals used in this study were of analytical grade.

### 2.2. Animals

Adult male SD rats (weighing 220 ± 10 g) were obtained from the Laboratory Animal Services Centre, The Chinese University of Hong Kong. The animals were maintained on a 12 h light/dark cycle under thermo-regulated (22 ± 2°C) and humidity-controlled (50 ± 10%) condition and provided standard diet and water *ad libitum*. The experimental procedures of the project were approved by the Animal Experimentation Ethics Committee of The Chinese University of Hong Kong (Ref. No. 18/264/MIS).

### 2.3. STZ Injection and Drug Treatment

Rats were anesthetized with ketamine (75 mg/kg) and xylazine (10 mg/kg) and then fixed placed on a stereotaxic apparatus (Stoelting, Wood Dale, IL, USA). The scalp of the rat was incised to locate the bregma. Small burr holes (1 mm in diameter) were drilled, and 5 *μ*L STZ (3 mg/kg) was injected into both sides of the lateral ventricle with a microsyringe fitted with a 26-gauge needle at a rate of 0.5 *μ*L/min with the following coordinates: 0.8 mm posterior to the bregma, ±1.4 mm lateral to the sagittal line, and 4.0 mm ventral to the surface of the skull, according to the brain atlas of Paxinos and Watson [18]. The needle was withdrawn after remaining for 5 min in the injection position. The incision was closed with wound clips, and the animal was allowed to recover under a heat lamp. Control-operated rats have received the same surgical procedures except with injection of the same volume of the vehicle (0.9% normal saline). The STZ injection was conducted twice on day 1 and day 3, respectively, for each rat.

**Figure 2:**
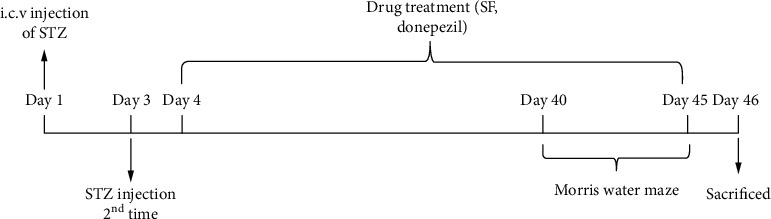
Experimental design and treatment schedule.

Twenty-four hours after the second STZ injection, the rats were randomly and equally divided into five groups (*n* = 8 per group) as follows: (1) control group, (2) STZ vehicle control group, (3) STZ+SF (25 mg/kg) group, (4) STZ+SF (50 mg/kg) group, and (5) STZ+donepezil (5 mg/kg) group. Both SF and donepezil were dissolved in ddH_2_O and given gavage with 10 mL/kg every day for 6 consecutive weeks. Rats in the control group and the STZ+vehicle control group were orally given an equal volume of ddH_2_O for 6 weeks. The MWMT was performed once a day for 4 days starting at day 40. During the MWMT, drug treatment was ongoing until the probe test, which was undertaken at day 45.  Figure 2 shows the experimental design and schedule. The treatment schedule and doses of SF were selected based on the results of our pilot study (data not shown).

### 2.4. MWMT

The MWMT was employed to test spatial learning and memory. Briefly, water was filled in a circular pool (150 cm diameter, 30 cm high) at 25°C. Four poles along the perimeter of the pool conceptually divided the maze into four equal quadrants. A hidden platform (10 cm in diameter, 26 cm in height) was placed 0.5 cm below the surface of the water and placed at the midpoint of one quadrant. The pool was located in a test room equipped with various prominent visual cues (e.g., pictures and lamps). Spatial training to find the hidden platform in the water maze was conducted for 4 consecutive days. On the first day of the spatial training, each rat was allowed to swim for a maximum of 60 sec three times, and then, each rat was assisted to the platform and allowed to rest for 30 sec each time. On the following days, each rat was trained three times per day; the rat was allowed to swim for a maximum of 60 sec each time. Rats that failed to find the platform within 60 sec were gently led to the platform, and the escape latency (finding the hidden platform) was recorded as 60 sec. The probe test was performed without the hidden platform on the 5^th^ day. Each rat was placed in the water and allowed to freely swim for 60 sec. The swimming routine, the swimming speed, and the time spent in the target quadrant were recorded and analyzed by using video tracking software SuperMaze V2.0 (Xinruan, Shanghai, China). Twenty-four hours after the MWMT, rats were sacrificed under deep anesthesia. The brains were rapidly harvested, and the hippocampus tissues were dissected. Each hippocampus was washed with cold sterile physiological saline and stored at -80°C until use.

### 2.5. Cell Culture and Treatment

The murine microglial BV-2 cell line was cultured in Roswell Park Memorial Institute 1640 Medium (RPMI 1640, Gibco, UT, USA) containing 10% fetal bovine serum (FBS, Gibco, CA, Brazil) and 1% penicillin-streptomycin (PS) at 37°C under an atmosphere of 5% CO_2_. BV-2 cells were seeded at a density of 5 × 10^3^ cells/well on a 96-well plate at 37°C overnight, unless otherwise specified. The cells were at first stabilized at 37°C for 24 h and subsequently incubated with different concentrations of SF (final concentrations: 0.5-32 *μ*M) for 1 h. LPS at a final concentration of 1 *μ*g/mL was then added to the culture for an additional 23 h. In the inhibitor test, LY294002 (5 *μ*M) was added 1 h prior to SF (2 *μ*M) treatment; then, cells were incubated for an additional 23 h.

### 2.6. Cell Viability Assay

The cytotoxicity of SF was measured by a quantitative colorimetric assay with the conventional MTT method. Briefly, BV-2 cells were seeded at a density of 5 × 10^3^ cells/well on a 96-well plate at 37°C overnight; then, the cells were treated with various concentrations of SF (0-32 *μ*M) for 24 h. After drug treatments, 20 *μ*L/well of MTT solution (final concentration of 1 mg/mL) was added, and cells were incubated at 37°C for 4 h. The supernatants were then aspirated off, and formazan crystals were dissolved with 150 *μ*L of DMSO. The optical density of each well was determined at 570 nm using a FLUOstar OPTIMA microplate reader (BMG Labtech, Offenburg, Germany). Cell viability was expressed as a percentage of the nontreated control.

### 2.7. Nitric Oxide (NO) Assay

The nitrite concentration in the culture medium was measured as an indicator of NO production according to the Griess reaction method [19]. Briefly, BV-2 cells were seeded onto a 24-well culture plate at a density of 2 × 10^4^ cells/well. The cell culture supernatants were collected after drug treatment. One hundred *μ*L of each supernatant was mixed with the same volume of Griess reagent (50 *μ*L 1% sulfanilamide in 5% phosphoric acid and 50 *μ*L 0.1% N-1-naphthylethylenediamine dihydrochloride in water). After incubation for 10 min at room temperature in the dark, the absorbance was measured at 540 nm using a microplate reader. Nitrite concentration was calculated using the standard curve of NaNO_2_.

### 2.8. Measurement of Cytokines

The levels of IL-6 (Cat. No. RK00008), TNF-*α* (Cat. No. RK00027), and IL-1*β* (Cat. No. RK00006) in the culture medium of BV-2 cell were measured by ELISA kits (ABclonal) according to the manufacturer's protocols. The concentration of each sample was calculated according to the standards provided in the kits. The levels of TNF-*α* and IL-10 were expressed as pg/mL.

Hippocampus samples were weighed and homogenized in a tenfold volume of lysis buffer. The homogenates were then centrifuged at 10,000 g for 30 min at 4°C, and the supernatants were used to determine the levels of TNF-*α* and IL-10. The total protein content was measured by using the BCA test. The level of IL-6 (Cat. No. ab234570) in the serum of rats and the levels of TNF-*α* (Cat. No. ab100785) and IL-10 (Cat. No. ab100765) in the hippocampus of rats were measured using ELISA kits (Abcam, Cambridge, UK) as per the manufacturer's instructions. The concentration of each sample was calculated according to the standards provided in the kits. The level of IL-6 was expressed as pg/mL, and the levels of TNF-*α* and IL-10 were expressed as pg/mg of protein.

### 2.9. Immunofluorescence Assay

BV-2 cells were immunostained to detect NF-*κ*B p65 location. In brief, the cells were plated on glass coverslips. After drug treatment, BV-2 cells were fixed in 4% paraformaldehyde, permeabilized with 1% Triton X-100, and blocked with 1% bovine serum albumin (BSA) in PBS. Next, the cells were incubated with primary antibodies against NF-*κ*B p65 (Cat. No. 6956S, Cell Signaling Technology) and p-NF-*κ*B p65 (Cat. No. 3033S, Cell Signaling Technology) overnight at 4°C. The cells were washed with PBS and incubated with fluorescence-conjugated secondary antibodies Alexa Fluor™ 647 Goat anti-mouse IgG (H+L) secondary antibody (Cat. No. A21235, Invitrogen) or Alexa Fluor 488 Donkey anti-Rabbit IgG (H+L) secondary antibody (Cat. No. R37118, Invitrogen) for 1 h at room temperature. The cells were mounted with ProLong™ Diamond Antifade Mountant with DAPI (Invitrogen).

The brains of the rats were removed and fixed by 4% paraformaldehyde. By using a frozen microtome, the brains were cut into 8 *μ*m sections. The sections were blocked in 5% BSA at room temperature for 20 min. Sections were incubated with anti-GFAP polyclonal antibody (Cat. No. HPA056030, Sigma) and anti-Iba-1 antibody (Cat. No. 019-19741, Wako) at 4°C overnight. Then, the sections were then incubated with fluorescence-conjugated secondary antibody Alexa Fluor 488 Donkey anti-Rabbit IgG (H+L) secondary antibody at room temperature for 1 h. Finally, the sections were mounted with ProLong™ Diamond Antifade Mountant with DAPI. Images were captured by using a Zeiss Axio Scope A1 microscope. The results were quantified by using ImageJ.

### 2.10. Western Blotting

Protein was extracted from the hippocampus of the rats or BV-2 cells by using RIPA buffer with an inhibitor of phosphorylase and protease. After extraction, the total protein content was measured using the BCA test. Protein samples were separated by SDS-PAGE and transferred to Polyvinylidene Fluoride (PVDF) membranes. Then, the membranes were blocked using nonfatty milk powder. The membranes were probed with the following primary antibodies: monoclonal rabbit anti-*β*-actin (Cat. No. 4967S), rabbit anti-GSK-3*β* (Cat. No. 9315S), rabbit anti-p-GSK-3*β* (Ser9) (Cat. No. 9336S), rabbit anti-p-Akt (S473) (Cat. No. 9271S), rabbit anti-Akt (Cat. No. 9272S), rabbit anti-PI3K p110*α* (Cat. No. 4249S, Cell Signaling), mouse anti-tau 46 (Cat. No. sc-32274), rabbit anti-p-tau (Thr205) (Cat. No. sc-101817) (Santa Cruz), rabbit anti-p-tau (Ser396) (Cat. No. ab109390), and rabbit anti-p-tau (Ser404) (Cat. No. ab92676) (Abcam), then incubated with a species-matched horseradish peroxidase-conjugated secondary antibody. The blots were visualized with ECL western blotting detection reagents (Invitrogen) and observed with Azure™ Biosystems c300. The optical density of immunoreactive bands was quantified using ImageJ.

### 2.11. Statistical Analysis

All values were represented as mean ± SEM. Data analyses were performed using the software of GraphPad Prism software (version 8, GraphPad Software, Inc., CA, USA). For the MWMT, escape latency in the hidden platform trial was analyzed with two-way ANOVA of repeated measures, with training days and drug treatments as variables. The other data were analyzed using one-way ANOVA followed by Dunnett's test to detect intergroup differences. A difference was considered significant at *p* < 0.05.

## 3. Results

### 3.1. Effects of SF on the Cognitive Impairments Induced by STZ in Rats

**Figure 3:**
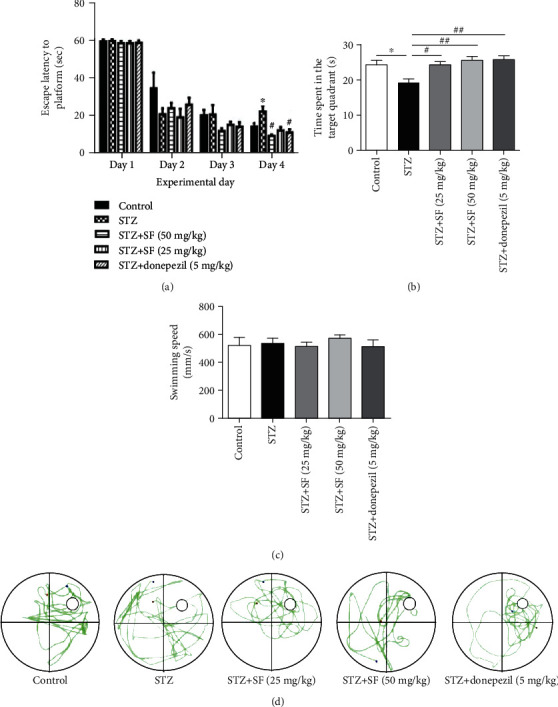
Effects of SF on the cognitive impairment induced by STZ in rats. (a) Escape latency analysis. (b) Time spent in the target quadrant in the probe test. (c) Swimming speed of different groups in the probe test. (d) Representative swimming routine of different groups in the probe test. The results are expressed as the mean ± SEM (*n* = 8). ^∗^*p* < 0.05 as compared with the control group; ^#^*p* < 0.05 and ^##^*p* < 0.01 as compared with the STZ-treated control group.

As shown in Figure 3(a), the performance of all groups of the rats was improved through training, as indicated by the shortened escape latency to the platform over consecutive days. A significant difference was found in the mean latency between training days (*F* (3, 140) = 261.9, *p* < 0.01) and between treatments (*F* (4, 140) = 3.456, *p* < 0.05), but no interaction was observed between training day and treatment (*F* (12, 140) = 1.679, *p* > 0.05). However, the STZ-treated rats took a longer time to find the platform throughout the training period compared to the control rats (day 2, *F* (4, 35) = 1.515, *p* > 0.05; day 3, *F* (4, 35) = 2.033, *p* > 0.05; day 4, *F* (4, 35) = 6.622, *p* < 0.01), suggesting that *i.c.v.* injection of STZ was able to cause cognitive impairment in rats.

In the probe test, time spent in the target quadrant in the STZ group was markedly decreased (*F* (4, 35) = 5.690, *p* < 0.01), as compared with the control group (Figure 3(b)). Administration of SF (25 and 50 mg/kg) significantly increased the time spent in the target quadrant (*p* < 0.05 and *p* < 0.01, respectively) in the STZ-treated rats, as compared to the STZ-treated control group. Donepezil (5 mg/kg) treatment also enhanced the time spent in the target quadrant (*p* < 0.01) by the STZ-treated rats, as compared to the STZ-treated control group. There was no significant difference in the swimming speeds of the rats from different groups (*F* (4, 35) = 2.137, *p* = 0.10) (Figure 3(c)). These data indicated that SF was able to significantly improve the learning and memory impairment induced by STZ.

### 3.2. Effects of SF on the STZ-Induced Neuroinflammation in Rats

**Figure 4:**
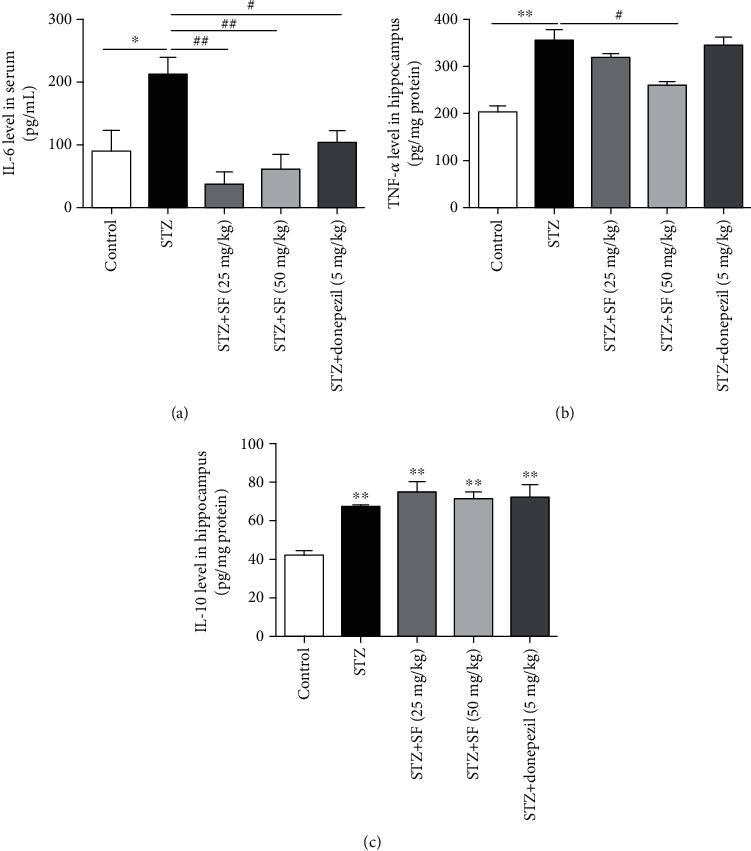
Effects of SF on the production of proinflammatory cytokines in the STZ-treated rats. (a) Concentration of IL-6 in the serum of the STZ-treated rats. (b, c) The levels of TNF-*α* and IL-10 in the hippocampus of the STZ-treated rats. The results are expressed as the mean ± SEM (*n* = 6). ^∗^*p* < 0.05 and ^∗∗^*p* < 0.01 as compared with the control group; ^#^*p* < 0.05 and ^##^*p* < 0.01 as compared with the STZ-treated control group.

As shown in Figures 4(a) and 4(b), the levels of TNF-*α* in the hippocampus and IL-6 in the serum were markedly increased (*F* (4, 25) = 15.04, *p* < 0.01 and *F* (4, 25) = 7.119, *p* < 0.01, respectively) in the STZ-treated rats. However, SF (25 and 50 mg/kg) treatment significantly reduced the release of TNF-*α* in the hippocampus (*p* > 0.05 and *p* < 0.01, respectively) and IL-6 in the serum (*p* < 0.01 for both) of the STZ-treated rats. On the other hand, the production of anti-inflammatory cytokine IL-10 was significantly upregulated (*F* (4, 25) = 10.71, *p* < 0.01) in the hippocampus of the STZ-treated rats, as compared with the control group. SF (25 and 50 mg/kg) treatment also significantly accentuated the level of IL-10 in the hippocampus of the STZ-treated rats, as compared with the control group, but no significant effects on the IL-10 production, as compared with the STZ-treated control group (Figure 4(c)).

### 3.3. Effects of SF on the Astrocytes and Microglia in the Hippocampus and Cerebral Cortex of the STZ-Treated Rats

**Figure 5:**
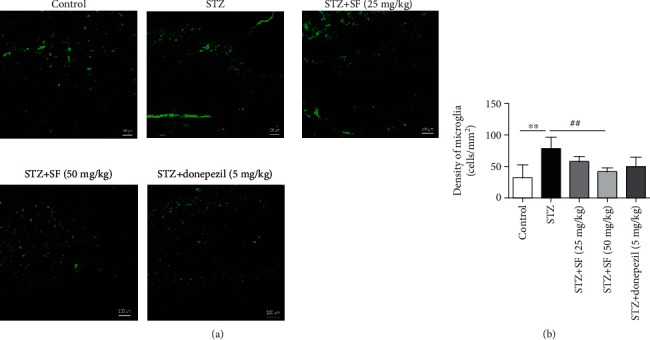
Effects of SF on Iba-1-positive microglia in the hippocampus of the STZ-treated rats. Microglia density was quantified by dividing the number of microglia by the area of the region of interest (cells/mm^2^). Data were expressed as mean ± SEM (*n* = 4). ^∗∗^*p* < 0.01 when compared with the control group; ^##^*p* < 0.01 when compared with the STZ-treated control group. The scale bar is 100 *μ*m in all figures.

**Figure 6:**
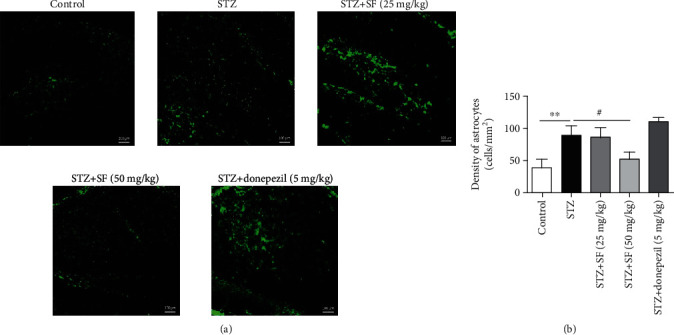
Effects of SF on GFAP-positive astrocytes in the hippocampus of the STZ-treated rats. Astrocyte density was quantified by dividing the number of astrocytes by the area of the region of interest (cells/mm^2^). Data were expressed as mean ± SEM (*n* = 4). ^∗∗^*p* < 0.01 when compared with the control group; ^#^*p* < 0.05 when compared with the STZ-treated control group. The scale bar was 100 *μ*m in all figures.

As shown in Figure 5, a significant increase of the Iba-1-positive microglia density was observed in the hippocampus (*F* (4, 15) = 5.434, *p* < 0.01) of the STZ-treated rats, when compared with the control group. SF treatment (50 mg/kg) significantly decreased the microglia density (*p* < 0.01) in the hippocampus of the STZ-treated rats, when compared with the STZ-treated control group (Figure 5(b)). Moreover, Figure 6 revealed that the GFAP-positive astrocyte density was markedly elevated (*F* (4, 15) = 17.57, *p* < 0.01) in the hippocampus of the STZ-treated rats, when compared with the control group. SF treatment (50 mg/kg) significantly decreased the astrocyte density in the hippocampus (*p* < 0.05), when compared with the STZ-treated control group (Figure 6(b)).

### 3.4. Effects of SF on the Hyperphosphorylation of Tau Protein in the Hippocampus of the STZ-Treated Rats

**Figure 7:**
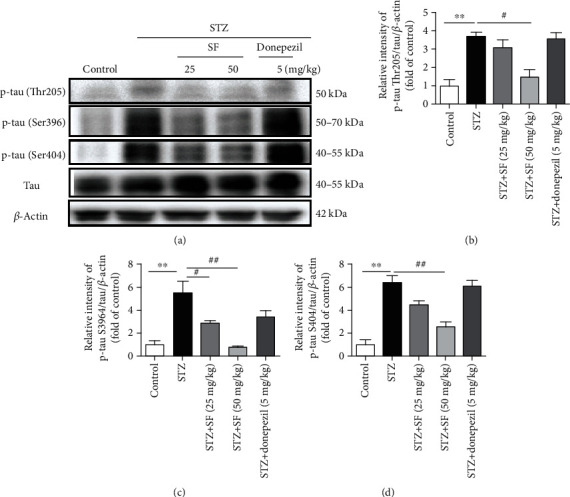
Effects of SF on the hyperphosphorylation of tau protein in the STZ-treated rats. (a) Representative immunoblot bands for p-tau (Thr205), p-tau (Ser396), p-tau (Ser404), and *β*-actin in rat hippocampus. (b–d) Quantitative analysis of immunoblot bands. Protein expression levels were normalized to *β*-actin. Data were expressed as mean ± SEM (*n* = 3). ^∗∗^*p* < 0.01 when compared with the control group; ^#^*p* < 0.05 and ^##^*p* < 0.01 when compared with the STZ group.

Alteration of tau phosphorylation in the hippocampus of the STZ-induced rats was investigated in this study. STZ injection significantly upregulated the protein expressions of phosphorylated tau protein (p-tau) at the Thr205, Ser396, and Ser404 sites in the hippocampus of the rats (Figure 7). The protein levels of p-tau (Thr205) (*F* (4, 10) = 11.36, *p* < 0.01), p-tau (Ser396) (*F* (4, 10) = 11.84, *p* < 0.01), and p-tau (Ser404) (*F* (4, 10) = 22.70, *p* < 0.01) in the hippocampus of the STZ-treated rats were significantly elevated, when compared with the control group (Figure 7). SF treatment (50 mg/kg) effectively inhibited the level of p-tau (Thr205) (*p* < 0.05) in the hippocampus of the STZ-treated rats, when compared to the STZ-treated control group. On the other hand, SF treatment (25 and 50 mg/kg) effectively suppressed the level of p-tau (Ser396) (*p* < 0.05 for both) in the hippocampus of the STZ-treated rats, when compared to the STZ-treated control group. Moreover, SF treatment (50 mg/kg) effectively reduced the level of p-tau (Ser404) (*p* < 0.01) in the hippocampus of the STZ-treated rats, when compared to the STZ-treated control group. The results amply demonstrated that SF plays an important role in blocking tau protein hyperphosphorylation.

### 3.5. Effects of SF on the PI3K/Akt/GSK-3*β* (Ser9) Pathway in the Hippocampus of the STZ-Treated Rats

**Figure 8:**
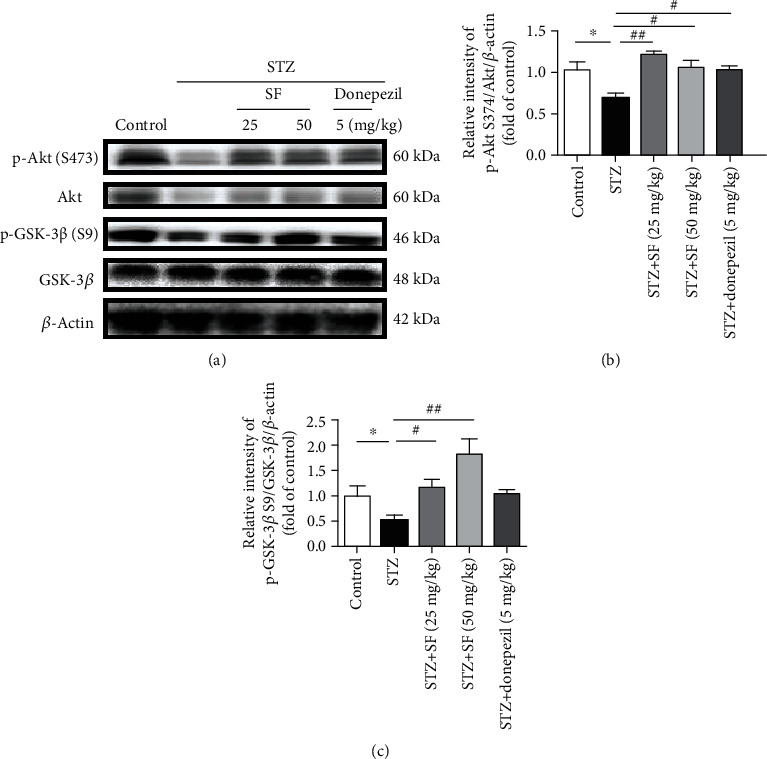
Effects of SF on the regulation of PI3K/Akt/GSK-3*β* in the STZ-treated rats. (a) Representative immunoblot bands for p-Akt (S473), Akt, p-GSK-3*β* (S9), GSK-3*β*, and *β*-actin in rat hippocampus. (b, c) Quantitative analysis of immunoblot bands. Protein expression levels were normalized to *β*-actin. The data were expressed as mean ± SEM (*n* = 3). ^∗^*p* < 0.05 and ^∗∗^*p* < 0.01 when compared with the control group; ^#^*p* < 0.05 and ^##^*p* < 0.01 when compared with the STZ-treated control group.

In this study, the phosphorylation levels of Akt (S473) and GSK-3*β* (S9) in the hippocampus of the rats were evaluated by western blotting. As shown in Figure 8, the ratios of p-Akt (S473)/Akt (*F* (4, 10) = 6.192, *p* < 0.01) and p-GSK-3*β* (S9)/GSK-3*β* (*F* (4, 10) = 7.666, *p* < 0.01) were markedly decreased in the hippocampus of the STZ-treated rats, as compared with the control group. However, SF treatment (25 and 50 mg/kg) effectively elevated the ratio of p-Akt/Akt (*p* < 0.01 and *p* < 0.05, respectively) in the hippocampus of the STZ-treated rats, when compared to the STZ-treated group (Figure 8(b)). Moreover, SF treatment (25 and 50 mg/kg) significantly accentuated the ratio of p-GSK-3*β* (S9)/GSK-3*β* (*p* < 0.05 and *p* < 0.01, respectively) in the hippocampus of the STZ-treated rats, when compared to the STZ-treated group (Figure 8(c)). These results indicate that SF may act as an activator of Akt, but as an inhibitor of the GSK-3*β* pathway.

### 3.6. Effect of SF on the Cytotoxicity and NO Production Induced by LPS in BV-2 Cells

**Figure 9:**
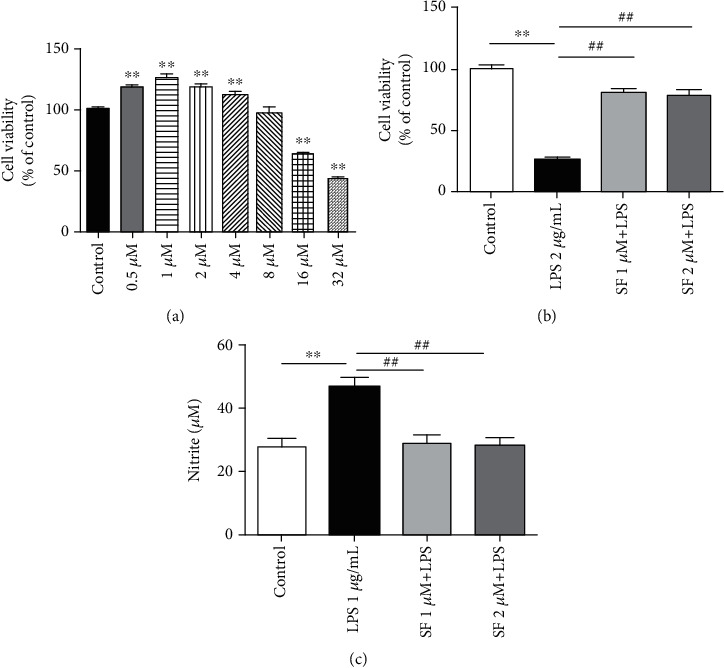
Effects of SF on the cytotoxicity and NO production in the LPS-stimulated BV-2 cells. (a) Cell viability of BV-2 cells treated with SF (0.5, 1, 2, 4, 8, 16, and 32 *μ*M) for 24 h. (b) The cell viability of BV-2 cells pretreated with SF (1 and 2 *μ*M) for 1 h followed by treatment with LPS (2 *μ*g/mL) for another 23 h. (c) The NO production of BV-2 cells pretreated with SF (1 and 2 *μ*M) for 1 h followed by treatment with LPS (1 *μ*g/mL) for another 23 h. Cell viability was assessed using the MTT assay. NO production was assessed using Griess reagent. The results are expressed as the mean ± SEM (*n* = 6). ^∗∗^*p* < 0.01 as compared with the control group; ^##^*p* < 0.01 as compared with the LPS-treated control group.

To explore the potential cytotoxicity of SF in BV-2 cells, MTT assay was performed. As shown in Figure 9(a), after treatment with SF at the doses ranging from 1 to 8 *μ*M for 24 h, there was no significant difference on the cell viability, as compared with the control group, indicating that SF was nontoxic to BV-2 cells at up to 8 *μ*M. Nontoxic concentrations (1 and 2 *μ*M) of SF were used in subsequent experiments. To ascertain the protective effects of SF in the LPS-stimulated BV-2 cells, cells were pretreated with SF (1 and 2 *μ*M) for 1 h followed by treatment with LPS (2 *μ*g/mL) for further 23 h. As demonstrated in Figure 8(b), LPS (2 *μ*g/mL) treatment significantly decreased the cell viability (*F* (3, 20) = 60.33, *p* < 0.01) in BV-2 cells, as compared with the control group. Pretreatment with SF (1 and 2 *μ*M) significantly attenuated the cell death (*p* < 0.01 for both) induced by LPS in BV-2 cells, as compared to the control group.

The potential anti-inflammatory property of SF was evaluated against the production of NO in the LPS-stimulated BV-2 cells. Based on the NO detection assay results, LPS alone markedly augmented the NO production (*F* (3, 20) = 12.86, *p* < 0.01), as compared to the control group (Figure 9(c)). However, pretreatment with SF (1 and 2 *μ*M) significantly reduced the level of NO (*p* < 0.01 for both) in the LPS-stimulated BV-2 cells, as compared with the LPS-treated control group.

### 3.7. Effects of SF on the Neuroinflammation Induced by LPS in BV-2 Cells

**Figure 10:**
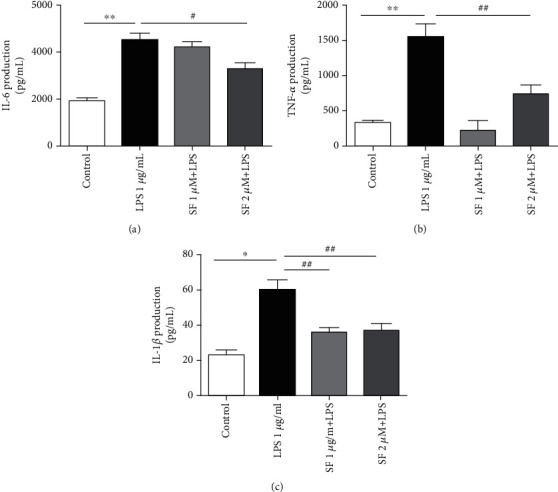
Effects of SF on the productions of (a) IL-6, (b) TNF-*α*, and (c) IL-1*β* in the LPS-stimulated BV-2 cells. The results are expressed as the mean ± SEM (*n* = 6). ^∗∗^*p* < 0.01 as compared with the control group; ^#^*p* < 0.05 and ^##^*p* < 0.01 as compared with the LPS-treated control group.

As shown in Figure 10, IL-6, TNF-*α*, and IL-1*β* levels were markedly increased (*F* (3, 20) = 25.92, *p* < 0.01; *F* (3, 20) = 24.50, *p* < 0.01; and *F* (3, 20) = 14.46, *p* < 0.01, respectively) in the culture medium of the LPS-stimulated BV-2 cells, as compared with the control group. However, pretreatment with SF (2 *μ*M) significantly reduced the levels of IL-6 (*p* < 0.05) and TNF-*α* (*p* < 0.01) in the LPS-stimulated BV-2 cells, as compared with the LPS-stimulated control group. On the other hand, pretreatment with SF (1 and 2 *μ*M) significantly reduced the levels of IL-1*β* (*p* < 0.01 for both) induced by LPS in BV-2 cells.

### 3.8. Effects of SF on the Activation of NF-*κ*B Induced by LPS in BV-2 Cells

**Figure 11:**
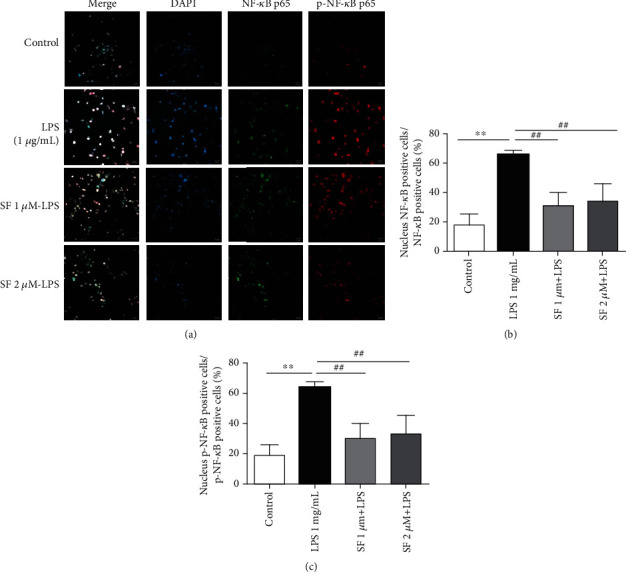
Effects of SF on the LPS-induced activation of NF-*κ*B in BV-2 cells. (a) Colocation of NF-*κ*B p65 (green) and p-NF-*κ*B p65 (red) with the nucleus (DAPI, blue) in BV-2 cells was analyzed by immunofluorescence. The scale bar is 20 *μ*m in all figures. (b, c) Quantitative analysis of NF-*κ*B p65 and p-NF-*κ*B p65 in the nuclei of BV-2 cells. The results were expressed as the mean ± SEM (*n* = 6). ^∗∗^*p* < 0.01 as compared with the control group; ^##^*p* < 0.01 as compared with the LPS-treated control group.

The colocation of NF-*κ*B p65 and p-NF-*κ*B p65 with the nucleus in BV-2 microglia was analyzed by immunofluorescence. Studies have reported that under inflammatory condition, NF-*κ*B is activated and transfers into the nucleus, then induces the productions of proinflammatory cytokines. As shown in Figure 11, both NF-*κ*B p65 and p-NF-*κ*B p65 were translocated into the nucleus in the LPS-stimulated BV-2 cells (*p* < 0.01 for both). However, pretreatment with SF (1 and 2 *μ*M) significantly suppressed the nuclear translocation of NF-*κ*B p65 and p-NF-*κ*B p65 in the LPS-stimulated BV-2 cells (*p* < 0.01 for both).

### 3.9. Effects of SF on the LPS-Induced Activation of GSK-3*β* in BV-2 Cells

**Figure 12:**
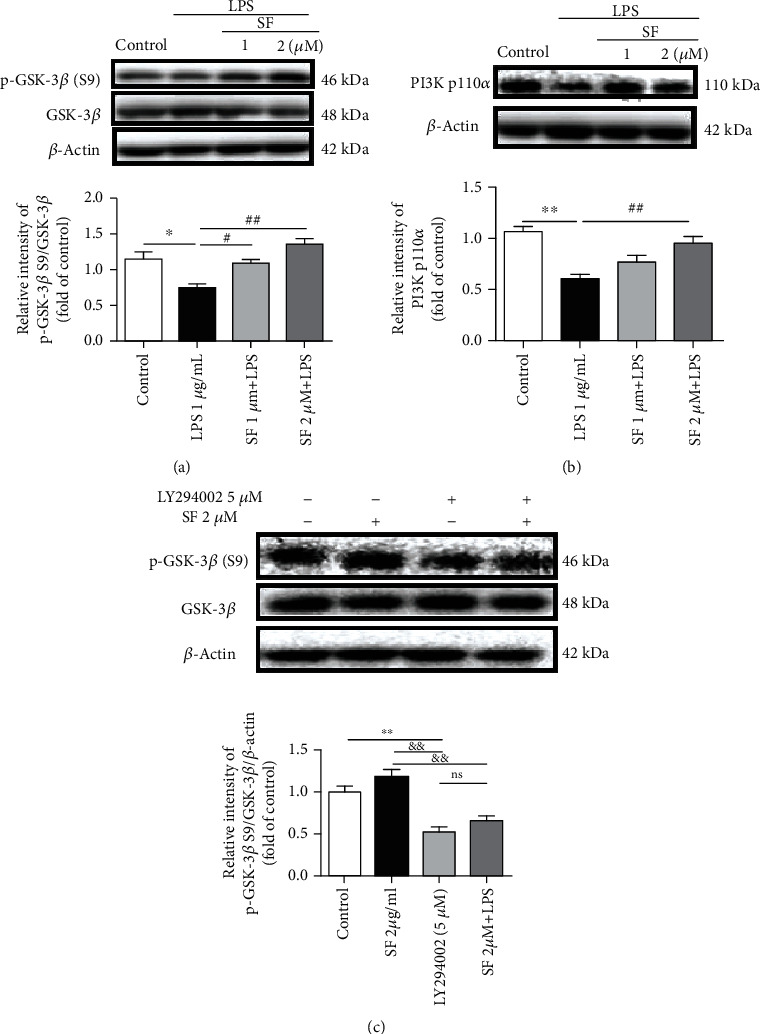
Effects of SF on the LPS-induced activation of GSK-3*β* and PI3K p110*α* by LPS in BV-2 cells. BV-2 cells were pretreated with SF (1 and 2 *μ*M) for 1 h followed by treatment with LPS (1 *μ*g/mL) for another 23 h. LY294002 (5 *μ*M) was pretreated for 1 h followed by treatment with SF (2 *μ*M) for another 23 h. (a) Representative immunoblot bands and quantitative analysis of p-GSK-3*β* (S9), GSK-3*β*, and *β*-actin in BV-2 cells. (b) Representative immunoblot bands and quantitative analysis of PI3K p110*α* and *β*-actin in BV-2 cells. (c) Representative immunoblot bands and quantitative analysis of p-GSK-3*β* (S9), GSK-3*β*, and *β*-actin in LY294002-treated BV-2 cells. The results were expressed as the mean ± SEM (*n* = 4). ^∗^*p* < 0.05 and ^∗∗^*p* < 0.01 as compared with the vehicle control group; ^#^*p* < 0.05 and ^##^*p* < 0.01 as compared with the LPS-treated control group; ^&&^*p* < 0.01 as compared with the SF treatment group; ns *p* > 0.05 as compared with the LY294002 group.

As shown in Figure 12, the protein expression of the ratio of p-GSK-3*β* (S9)/GSK-3*β* (*F* (3, 12) = 9.794, *p* < 0.01) and PI3K p110*α* (*F* (3, 12) = 5.315, *p* < 0.01) was significantly attenuated in the LPS-stimulated BV-2 cells, as compared with the control group. Pretreatment with SF (1 and 2 *μ*M) restored the protein expression of the ratio of p-GSK-3*β* (S9)/GSK-3*β* in the LPS-stimulated BV-2 cells (*p* < 0.05 and *p* < 0.01, respectively). Pretreatment with SF (2 *μ*M) restored the protein expression of PI3K p110*α*, as compared with the LPS-stimulated group (*p* < 0.01). Pretreated LY294002 showed significantly reduced levels of p-GSK-3*β* (S9) (*F* (3, 12) = 15.74, *p* < 0.01). Moreover, the effect of SF on p-GSK-3*β* (S9) was blocked by LY294002 (*p* < 0.01). These results indicate that the effects of SF on BV-2 cells depend on the PI3K/Akt/GSK-3*β* pathway.

## 4. Discussion

In recent years, SF has attracted increasing attention due to its low toxicity; its known antitumor [20–23], antibacterial, and antiviral properties [24, 25]; its ability in regulating the skewed gut microbiota; and its protective effect in inflammatory bowel disease [26]. However, few studies have reported the protective effects of SF on the central nervous system (CNS). In this study, we reported for the first time that SF ameliorated cognitive deficits induced by STZ in rats through inhibition of neuroinflammation and tau protein hyperphosphorylation via regulating the PI3K/Akt/GSK-3*β* pathway. This study provides new insights into the potential of SF as a promising therapeutic candidate for AD treatment.

Neuroinflammation is the innate immune response in the CNS that involves neurons, astrocytes, and microglia to protect the CNS against infection and injury [27]. However, increasing evidence has indicated that neuroinflammation is central to the common pathology of several acute and chronic brain diseases, especially AD [28]. Under neuroinflammation condition, microglia and astrocytes are activated and then cause the release of inflammatory cytokines such as TNF-*α*, IL-6, and IL-1*β*, resulting in synapse dysfunction and neuronal death in AD patients [29, 30]. Studies have demonstrated a marked augmentation of the levels of TNF-*α*, IL-6, and IL-1*β* in the serum and brains of AD patients and AD animal models [31–35]. The increase in proinflammatory levels facilitates the A*β* synthase [36] and tau protein phosphorylation [37]. Thus, many anti-inflammation drugs have been investigated to prevent or treat AD such as nonsteroidal anti-inflammatory drugs (NSAIDs) and aspirin [38, 39]. In the present study, SF treatment significantly inhibited the level of IL-6 in serum and the levels of TNF-*α* in the hippocampus of the STZ-treated rats. Moreover, to elucidate the molecular mechanisms underlying the antineuroinflammatory effects of SF, we further investigated the inhibitory effects of SF on the productions of NO, TNF-*α*, IL-6, and IL-1*β* in the LPS-stimulated BV-2 microglial cells. Our results indicated that SF was able to significantly inhibit the levels of NO, TNF-*α*, and IL-6 induced by LPS in BV-2 cells. These results indicate that the cognitive deficits improving the effect of SF are, at least partially, related to its anti-inflammatory property.

Microglia are the CNS resident macrophages widely distributed in the brain. Reactive astrocytes are characterized by the increased expression of glial fibrillary acidic protein (GFAP) [40], while Iba-1 plays as a unique marker for detecting microglial activation [41]. Activated microglia and astrocyte are known to be associated with A*β* deposition and NFT accumulation during the progress of AD [42, 43]. Our immunofluorescence assays showed that SF administration diminished the activation of microglia (Iba-1) and astrocytes (GFAP) in the hippocampus of the STZ-induced rats.

NF-*κ*B is a predominant transcription factor responsible for the regulation of microglia-mediated neuroinflammation [27]. Generally, NF-*κ*B is located within the cytoplasm and kept inactive by interaction with its inhibitor. Once activated, phosphorylated NF-*κ*B dimers are released and translocated to the nucleus, which induces the production of cytokines, inducible nitric oxide synthase (iNOS), and NO in microglia [44–46]. Previous studies have reported that the level of NF-*κ*B p65 in the brains of AD patients was significantly elevated [47]. Moreover, the increase of NF-*κ*B activity has been observed in AD mouse models with neuroapoptosis [48]. Therefore, inhibition of the activation of NF-*κ*B in microglia could regulate the progression of AD caused by neuroinflammation. Our results also showed that SF treatment was able to inhibit the nuclear translocation of p-NF-*κ*B p65 induced by LPS in BV-2 cells. These findings amply indicated that SF inhibited the activation of the NF-*κ*B pathway to suppress the neuroinflammation induced by LPS in BV-2 cells.

Tau, the major microtubule-associated protein (MAP) of normal mature neurons, is essential in the assembly as well as stabilization of the structural integrity of microtubules [49]. In the AD brain, tau protein is more hyperphosphorylated than normal neurons, loses its ability to bind to microtubules, and subsequently polymerizes into paired helical filaments admixed with straight filaments forming neurofibrillary tangles [50]. A significant increase in the phosphorylation of tau protein at Thr205, Ser396, and Ser404 is observed in the brains of AD patients [51]. The induction of tau phosphorylation at Ser404 and Ser396 has been reported to relate to oxidative stress which is one of the earliest events in AD [52, 53]. Tau protein phosphorylation at the Thr205 site is sufficient to induce NFT aggregation without the addition of any exogenous aggregation inducer [54]. In our study, SF treatment significantly downregulated the protein expressions of p-tau at Thr205, Ser396, and Ser404 sites in the hippocampus of the STZ-treated rats. Our results indicated that the inhibitory effect of SF on these specific hyperphosphorylation sites of tau protein is one of the action mechanisms underlying its cognitive deficits improving the effect.

**Figure 13:**
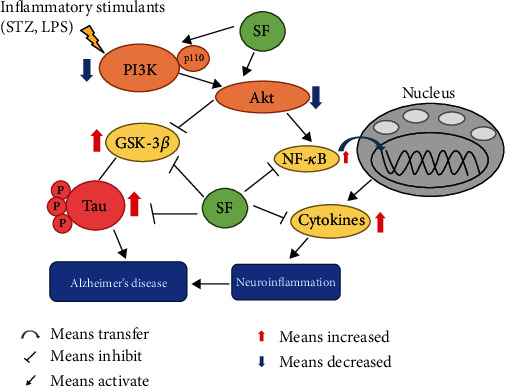
The schematic drawing illustrating the action mechanisms underlying the effects of SF on ameliorating the cognitive impairments induced by STZ in rats. Our findings revealed that SF was able to suppress the activation of microglia and astrocytes to reduce the release of proinflammatory cytokines such as IL-6 and TNF-*α*, while promoting the production of the anti-inflammatory mediator such as IL-10. SF also inhibited tau protein phosphorylation by modulating the PI3K/Akt/GSK-3*β* pathway. Moreover, SF ameliorated neuroinflammation stimulated by LPS in BV-2 cells through inhibiting the NF-*κ*B p65 translocation to the nucleus and regulating the PI3K/Akt/GSK-3*β* pathway. All these molecular actions of SF collectively contribute to its therapeutic effects on the experimental models of AD.

The PI3K/Akt signaling pathway is one of the most important pathways for neuron survival and plays a pivotal role in neuroinflammation [55]. PI3K is a heteromeric protein consisting of a p110 catalytic subunit and a p85 regulatory subunit. PI3K p110 subunits convert phosphatidylinositol (3,4)-bisphosphate (PIP2) into phosphatidylinositol (3,4,5)-trisphosphate (PIP3), which in turn activates the downstream kinases, such as Akt [56]. Generally, to complete the full activation of Akt, phosphorylation is needed at the Ser473 site [57, 58]. Glycogen synthase kinase-3*β* (GSK-3*β*), a downstream kinase of the PI3K/Akt signaling pathway, is a constitutively active protein kinase with a number of biological functions involving neuroinflammation and aggregation of NFTs [59, 60]. When activated, GSK-3*β* promotes microglial migration and inflammatory activation [61] through inducing the production of proinflammatory cytokines via NF-*κ*B [62] and NO [63] in microglia. Moreover, activated GSK-3*β* promotes the phosphorylation of tau protein at multiple sites [64]. GSK-3*β* is rendered inactive when it is phosphorylated at Ser9 by activated Akt. Previous studies have reported that the levels of PI3K subunits (both p85 and p110) and phosphorylations of Akt at the Ser473 site and GSK-3*β* at the Ser9 site are decreased in the postmortem AD brain samples [65, 66]. Our results revealed that SF ameliorated the downregulation of PI3K p110*α* induced by LPS in BV-2 cells. On the other hand, SF treatment upregulated the protein expression of the ratio of p-Akt (Ser473)/Akt in the hippocampus of the STZ-treated rats. Moreover, SF inhibited the activation of GSK-3*β* induced by LPS in BV-2 microglia cells and in the STZ-treated rats via increasing the protein expression of the ratio of p-GSK-3*β* (Ser9)/GSK-3*β*. In addition, LY294002 is a selective PI3K inhibitor, which acts by competitively inhibiting the ATP-binding site of PI3K [67]. From our present results, LY294002 effectively abrogated the effects of SF on p-GSK-3*β* (Ser9) expression, which indicated that the PI3K/Akt signaling pathway was involved in the regulation of GSK-3*β* phosphorylation. These results indicated that SF improved cognitive function and reduced the AD pathology via modulating the PI3K/Akt/GSK-3*β* signaling pathway.  Figure 13 provides a summary on the action mechanisms underlying the effects of SF on ameliorating the cognitive impairments induced by STZ in rats.

## 5. Conclusion

In summary, our results demonstrated that SF, a Raphani Semen-derived isothiocyanate, was able to ameliorate cognitive impairments induced by STZ in rats partially via inhibiting neuroinflammation and hyperphosphorylation of tau protein through modulating the PI3K/Akt/GSK-3*β* pathway. These findings provide scientific evidence for the potential application of SF and Raphani Semen for the clinical treatment of AD. Further studies are ongoing in our laboratories to determine the pharmacokinetics and toxicity profiles of SF using relevant experimental AD models.

## Data Availability

All data supporting the conclusions of this article are included in this article.

## Abbreviations

A*β*: *β*-Amyloid peptide
AD: Alzheimer's disease
Akt: Protein kinase B
CNS: Central nervous system
GFAP: Glial fibrillary acidic protein
GSK-3*β*: Glycogen synthase kinase-3*β*
i.c.v.: Intracerebroventricular
IL-6: Interleukin-6
IL-1*β*: Interleukin-1*β*
IL-10: Interleukin-10
LPS: Lipopolysaccharide
MTT: 3-(4,5-Dimethyl-2-thiazolyl)-2,5-diphenyl-2-H-tetrazolium bromide
MWMT: Morris Water Maze Test
NF-*κ*B: Nuclear factor kappa-B
NFTs: Neurofibrillary tangles
NO: Nitric oxide
PI3K: Phosphoinositide 3-kinase
Ser/S: Serine
SF: Sulforaphene
STZ: Streptozotocin
Thr: Threonine
TNF-*α*: Tumor necrosis factor-*α*.

## References

[1] Blennow K., de Leon M. J., Zetterberg H. (2006). Alzheimer’s disease. *The Lancet*.

[2] Wang W. Y., Tan M. S., Yu J. T., Tan L. (2015). Role of pro-inflammatory cytokines released from microglia in Alzheimer’s disease. *Annals of Translational Medicine*.

[3] Ravelli K. G., Rosário B. . A., Camarini R., Hernandes M. S., Britto L. R. (2017). Intracerebroventricular streptozotocin as a model of Alzheimer’s disease: neurochemical and behavioral characterization in mice. *Neurotoxicity Research*.

[4] Bao J., Mahaman Y. A. R., Liu R. (2017). Sex differences in the cognitive and hippocampal effects of streptozotocin in an animal model of sporadic AD. *Frontiers in Aging Neuroscience*.

[5] Wu C., Yang L., Tucker D. (2018). Beneficial effects of exercise pretreatment in a sporadic Alzheimer’s rat model. *Medicine and Science in Sports and Exercise*.

[6] Biasibetti R., Almeida dos Santos J. P., Rodrigues L. (2017). Hippocampal changes in STZ-model of Alzheimer’s disease are dependent on sex. *Behavioural Brain Research*.

[7] Sharma M., Gupta Y. K. (2001). Intracerebroventricular injection of streptozotocin in rats produces both oxidative stress in the brain and cognitive impairment. *Life Sciences*.

[8] Salkovic-Petrisic M., Knezovic A., Hoyer S., Riederer P. (2013). What have we learned from the streptozotocin-induced animal model of sporadic Alzheimer’s disease, about the therapeutic strategies in Alzheimer’s research. *Journal of Neural Transmission*.

[9] Dinkova-Kostova A. T., Kostov R. V. (2012). Glucosinolates and isothiocyanates in health and disease. *Trends in Molecular Medicine*.

[10] Zhang R., Zhang J., Fang L. (2014). Neuroprotective effects of sulforaphane on cholinergic neurons in mice with Alzheimer’s disease-like lesions. *International Journal of Molecular Sciences*.

[11] Kim H. V., Kim H. Y., Ehrlich H. Y., Choi S. Y., Kim D. J., Kim Y. (2012). Amelioration of Alzheimer’s disease by neuroprotective effect of sulforaphane in animal model. *Amyloid*.

[12] Lee S., Choi B. R., Kim J. (2018). Sulforaphane Upregulates the Heat Shock Protein Co-Chaperone CHIP and Clears Amyloid-*β* and Tau in a Mouse Model of Alzheimer's Disease. *Molecular Nutrition & Food Research*.

[13] Pu D., Zhao Y., Chen J. (2018). Protective effects of sulforaphane on cognitive impairments and AD-like lesions in diabetic mice are associated with the upregulation of Nrf2 transcription activity. *Neuroscience*.

[14] Hou T. T., Yang H. Y., Wang W., Wu Q. Q., Tian Y. R., Jia J. P. (2018). Sulforaphane inhibits the generation of amyloid-*β* oligomer and promotes spatial learning and memory in Alzheimer’s disease (PS1V97L) transgenic mice. *Journal of Alzheimer’s Disease*.

[15] Ivánovics G., Horváth S. (1947). Raphanin, an antibacterial principle of the radish (Raphanus sativus). *Nature*.

[16] Liang H., Yuan Q., Dong H., Qian Z., Liu Y. (2004). Comparison of sulforaphane content in seeds of cruciferous plant. *Chinese Pharmaceutical Journal*.

[17] Kuang P., Song D., Yuan Q., Lv X., Zhao D., Liang H. (2013). Preparative separation and purification of sulforaphene from radish seeds by high-speed countercurrent chromatography. *Food Chemistry*.

[18] Paxinos G., Watson C. (1998). *The rat brain in stereotaxic coordinates*.

[19] Xian Y. F., Li Y. C., Ip S. P., Lin Z. X., Lai X. P., Su Z. R. (2011). Anti-inflammatory effect of patchouli alcohol isolated from Pogostemonis Herba in LPS-stimulated RAW264.7 macrophages. *Experimental and Therapeutic Medicine*.

[20] Yang M., Wang H., Zhou M. (2016). The natural compound sulforaphene, as a novel anticancer reagent, targeting PI3K-AKT signaling pathway in lung cancer. *Oncotarget*.

[21] Pawlik A., Wala M., Hac A., Felczykowska A., Herman-Antosiewicz A. (2017). Sulforaphene, an isothiocyanate present in radish plants, inhibits proliferation of human breast cancer cells. *Phytomedicine*.

[22] Kntayya S. B., Ibrahim M. D., Mohd Ain N., Iori R., Ioannides C., Abdull Razis A. F. (2018). Induction of apoptosis and cytotoxicity by isothiocyanate sulforaphene in human hepatocarcinoma HepG2 cells. *Nutrients*.

[23] Wang H., Wang F., Wu S. (2018). Traditional herbal medicine-derived sulforaphene promotes mitophagic cell death in lymphoma cells through CRM1-mediated p62/SQSTM1 accumulation and AMPK activation. *Chemico-Biological Interactions*.

[24] Gutiérrez R. M. P., Perez R. L. (2004). *Raphanus sativus* (radish): their chemistry and biology. *Scientific World Journal*.

[25] Lim S., Han S. W., Kim J. (2016). Sulforaphene identified from radish (*Raphanus sativus* L.) seeds possesses antimicrobial properties against multidrug-resistant bacteria and methicillin-resistant *Staphylococcus aureus*. *Journal of Functional Foods*.

[26] Li M., Gao J., Tang Y. (2017). Traditional herbal medicine-derived sulforaphene LFS-01 reverses colitis in mice by selectively altering the gut microbiota and promoting intestinal gamma-delta T cells. *Frontiers in Pharmacology*.

[27] Zhang F., Jiang L. (2015). Neuroinflammation in Alzheimer’s disease. *Neuropsychiatric Disease and Treatment*.

[28] Lyman M., Lloyd D. G., Ji X., Vizcaychipi M. P., Ma D. (2014). Neuroinflammation: the role and consequences. *Neuroscience Research*.

[29] Domingues C., da Cruz e Silva O. A. B., Henriques A. G. (2017). Impact of cytokines and chemokines on Alzheimer’s disease neuropathological hallmarks. *Current Alzheimer Research*.

[30] Heneka M. T., Carson M. J., Khoury J. E. (2015). Neuroinflammation in Alzheimer’s disease. *The Lancet Neurology*.

[31] Gezen-Ak D., Dursun E., Hanağası H. (2013). BDNF, TNF*α*, HSP90, CFH, and IL-10 serum levels in patients with early or late onset Alzheimer’s disease or mild cognitive impairment. *Journal of Alzheimer’s Disease*.

[32] McAlpine F. E., Lee J. K., Harms A. S. (2009). Inhibition of soluble TNF signaling in a mouse model of Alzheimer’s disease prevents pre-plaque amyloid-associated neuropathology. *Neurobiology of Disease*.

[33] Ghosh S., Wu M. D., Shaftel S. S. (2013). Sustained interleukin-1*β* overexpression exacerbates tau pathology despite reduced amyloid burden in an Alzheimer’s mouse model. *Journal of Neuroscience*.

[34] Batarseh Y., Duong Q.-V., Mousa Y., Al Rihani S., Elfakhri K., Kaddoumi A. (2016). Amyloid-*β* and astrocytes interplay in Amyloid-*β* related disorders. *International Journal of Molecular Sciences*.

[35] Gonzalez-Reyes R. E., Nava-Mesa M. O., Vargas-Sanchez K., Ariza-Salamanca D., Mora-Munoz L. (2017). Involvement of astrocytes in Alzheimer’s disease from a neuroinflammatory and oxidative stress perspective. *Frontiers in Molecular Neuroscience*.

[36] Ghosal K., Vogt D. L., Liang M., Shen Y., Lamb B. T., Pimplikar S. W. (2009). Alzheimer’s disease-like pathological features in transgenic mice expressing the APP intracellular domain. *Proceedings of the National Academy of Sciences of the United States of America*.

[37] Krstic D., Madhusudan A., Doehner J. (2012). Systemic immune challenges trigger and drive Alzheimer-like neuropathology in mice. *Journal of Neuroinflammation*.

[38] Brenneis C., Coste O., Altenrath K. (2011). Anti-inflammatory role of microsomal prostaglandin E synthase-1 in a model of neuroinflammation. *The Journal of Biological Chemistry*.

[39] De Jong D., Jansen R., Hoefnagels W. (2008). No effect of one-year treatment with indomethacin on Alzheimer’s disease progression: a randomized controlled trial. *PLoS One*.

[40] Olabarria M., Noristani H. N., Verkhratsky A., Rodríguez J. J. (2011). Age-dependent decrease in glutamine synthetase expression in the hippocampal astroglia of the triple transgenic Alzheimer’s disease mouse model: mechanism for deficient glutamatergic transmission?. *Molecular Neurodegeneration*.

[41] Imai Y., Ibata I., Ito D., Ohsawa K., Kohsaka S. (1996). A novel gene iba1 in the major histocompatibility complex class III region encoding an EF hand protein expressed in a monocytic lineage. *Biochemical and Biophysical Research Communications*.

[42] Hartlage-Rübsamen M., Zeitschel U., Apelt J. (2003). Astrocytic expression of the Alzheimer’s disease *β*-secretase (BACE1) is stimulus-dependent. *Glia*.

[43] Lee D. C., Rizer J., Selenica M. L. B. (2010). LPS- induced inflammation exacerbates phospho-tau pathology in rTg4510 mice. *Journal of Neuroinflammation*.

[44] Jana M., Dasgupta S., Liu X., Pahan K. (2002). Regulation of tumor necrosis factor-alpha expression by CD40 ligation in BV-2 microglial cells. *Journal of Neurochemistry*.

[45] Nakajima K., Matsushita Y., Tohyama Y., Kohsaka S., Kurihara T. (2006). Differential suppression of endotoxin-inducible inflammatory cytokines by nuclear factor kappa B (NF*κ*B) inhibitor in rat microglia. *Neuroscience Letters*.

[46] Bhat N. R., Feinstein D. L., Shen Q., Bhat A. N. (2002). p38 MAPK-mediated transcriptional activation of inducible nitric-oxide synthase in glial cells. Roles of nuclear factors, nuclear factor kappa B, cAMP response element-binding protein, CCAAT/enhancer-binding protein-beta, and activating transcription factor-2. *The Journal of Biological Chemistry*.

[47] Chen C. H., Zhou W., Liu S. (2012). Increased NF-*κ*B signalling up-regulates BACE1 expression and its therapeutic potential in Alzheimer’s disease. *The International Journal of Neuropsychopharmacology*.

[48] Niu Y. L., Zhang W. J., Wu P. (2010). Expression of the apoptosis-related proteins caspase-3 and NF-kappaB in the hippocampus of Tg2576 mice. *Neuroscience Bulletin*.

[49] Kiris E., Ventimiglia D., Feinstein S. C. (2010). Quantitative analysis of MAP-mediated regulation of microtubule dynamic instability in vitro. *Methods in Cell Biology*.

[50] Iqbal K., Liu F., Gong C. X., Grundke-Iqbal I. (2010). Tau in Alzheimer disease and related tauopathies. *Current Alzheimer Research*.

[51] Zhou X. W., Li X., Bjorkdahl C. (2006). Assessments of the accumulation severities of amyloid *β*-protein and hyperphosphorylated tau in the medial temporal cortex of control and Alzheimer’s brains. *Neurobiology of Disease*.

[52] Gomez-Ramos A., Diaz-Nido J., Smith M. A., Perry G., Avila J. (2003). Effect of the lipid peroxidation product acrolein on tau phosphorylation in neural cells. *Journal of Neuroscience Research*.

[53] Mondragon-Rodriguez S., Perry G., Luna-Munoz J., Acevedo-Aquino M. C., Williams S. (2014). Phosphorylation of tau protein at sites Ser(396-404) is one of the earliest events in Alzheimer’s disease and Down syndrome. *Neuropathology and Applied Neurobiology*.

[54] Despres C., Byrne C., Qi H. (2017). Identification of the tau phosphorylation pattern that drives its aggregation. *Proceedings of the National Academy of Sciences of the United States of America*.

[55] Shabab T., Khanabdali R., Moghadamtousi S. Z., Kadir H. A., Mohan G. (2016). Neuroinflammation pathways: a general review. *The International Journal of Neuroscience*.

[56] Boura-Halfon S., Zick Y. (2009). Chapter 12 serine kinases of insulin receptor substrate proteins. *Vitamins Hormones*.

[57] Kitagishi Y., Nakanishi A., Minami A. (2014). Certain diet and lifestyle may contribute to islet *β*-cells protection in type-2 diabetes via the modulation of cellular PI3K/AKT pathway. *The Open Biochemistry Journal*.

[58] Kitagishi Y., Nakanishi A., Ogura Y., Matsuda S. (2014). Dietary regulation of PI3K/AKT/GSK-3*β* pathway in Alzheimer’s disease. *Alzheimer’s Research & Therapy*.

[59] Su H. C., Ma C. T., Yu B. C. (2012). Glycogen synthase kinase-3*β* regulates anti-inflammatory property of fluoxetine. *International Immunopharmacology*.

[60] Lee C. W. C., Lau K. F., Miller C. C. J., Shaw P. C. (2003). Glycogen synthase kinase-3*β*-mediated tau phosphorylation in cultured cell lines. *Neuroreport*.

[61] Yuskaitis C. J., Jope R. S. (2009). Glycogen synthase kinase-3 regulates microglial migration, inflammation, and inflammation-induced neurotoxicity. *Cellular Signalling*.

[62] Wang M. J., Huang H. Y., Chen W. F., Chang H. F., Kuo J. S. (2010). Glycogen synthase kinase-3*β* inactivation inhibits tumor necrosis factor-*α* production in microglia by modulating nuclear factor *κ*B and MLK3/JNK signaling cascades. *Journal of Neuroinflammation*.

[63] Huang W. C., Lin Y. S., Wang C. Y. (2009). Glycogen synthase kinase-3 negatively regulates anti-inflammatory interleukin-10 for lipopolysaccharide-induced iNOS/NO biosynthesis and RANTES production in microglial cells. *Immunology*.

[64] Martin L., Page G., Terro F. (2011). Tau phosphorylation and neuronal apoptosis induced by the blockade of PP2A preferentially involve GSK3*β*. *Neurochemistry International*.

[65] Steen E., Terry B. M., Rivera E. J. (2005). Impaired insulin and insulin-like growth factor expression and signaling mechanisms in Alzheimer’s disease--is this type 3 diabetes?. *Journal of Alzheimer’s Disease*.

[66] Moloney A. M., Griffin R. J., Timmons S., O’Connor R., Ravid R., O’Neill C. (2010). Defects in IGF-1 receptor, insulin receptor and IRS-1/2 in Alzheimer’s disease indicate possible resistance to IGF-1 and insulin signalling. *Neurobiology of Aging*.

[67] Vlahos C. J., Matter W. F., Hui K. Y., Brown R. F. (1994). A specific inhibitor of phosphatidylinositol 3-kinase, 2-(4-morpholinyl)-8-phenyl-4H-1-benzopyran-4-one (LY294002). *The Journal of Biological Chemistry*.

